# Factor Graph-Assisted Distributed Cooperative Positioning Algorithm in the GNSS System

**DOI:** 10.3390/s18113748

**Published:** 2018-11-02

**Authors:** Chengkai Tang, Lingling Zhang, Yi Zhang, Houbing Song

**Affiliations:** 1 School of Electronics and Information, Northwestern Polytechnical University, Xi’an 710072, China; cktang@nwpu.edu.cn (C.T.); zhangyi@nwpu.edu.cn (Y.Z.); 2 School of Marine Science and Technology, Northwestern Ploytechnical University, Xi’an 710072, China; 3 Department of Electrical, Computer, Software, and Systems Engineering, Embry-Riddle Aeronautical University, Daytona Beach, FL 32114, USA; Houbing.Song@erau.edu; 4 Shaanxi Key Laboratory of Integrated and Intelligent Navigation, Xi’an 710000, China

**Keywords:** cooperative positioning, distributed positioning, factor graphs, total least squares

## Abstract

The development of smart cities calls for improved accuracy in navigation and positioning services; due to the effects of satellite orbit error, ionospheric error, poor quality of navigation signals and so on, it is difficult for existing navigation technology to achieve further improvements in positioning accuracy. Distributed cooperative positioning technology can further improve the accuracy of navigation and positioning with existing GNSS (Global Navigation Satellite System) systems. However, the measured range error and the positioning error of the cooperative nodes exhibit larger reductions in positioning accuracy. In response to this question, this paper proposed a factor graph-aided distributed cooperative positioning algorithm. It establishes the confidence function of factor graphs theory with the ranging error and the positioning error of the coordinated nodes and then fuses the positioning information of the coordinated nodes by the confidence function. It can avoid the influence of positioning error and ranging error and improve the positioning accuracy of cooperative nodes. In the simulation part, the proposed algorithm is compared with a mainly coordinated positioning algorithm from four aspects: the measured range error, positioning error, convergence speed, and mutation error. The simulation results show that the proposed algorithm leads to a 30–60% improvement in positioning accuracy compared with other algorithms under the same measured range error and positioning error. The convergence rate and mutation error elimination times are only 1/5 to 1/3 of the other algorithms.

## 1. Introduction

With the development of smart cities, navigation and positioning techniques are now more important in daily life. However, it is extremely difficult to improve the accuracy of navigation and position with existing satellite navigation systems, Inertial Navigation Systems (INS), and other navigation systems. The services required by smart cities, such as autonomous vehicle driving and unmanned aerial vehicles, require the support of high-precision navigation and positioning services. The existing navigation and positioning technology mainly improves the accuracy of navigation and positioning by improving the signal quality of satellite navigation systems [1], enhancing navigation signal strength [2], map matching [3], ground station assistance [4], and so on. These techniques can significantly improve positioning accuracy in sparsely populated areas. In urban environments, it is difficult to substantially improve the positioning accuracy in ill-conditioned wireless environments due to the multipath effect and the high-rise effect of the building. However, it is theoretically feasible to improve positioning accuracy through cooperative locations among multi-user terminals due to the large number of terminals. A simple cooperative positioning method based on a wireless sensor network was proposed by Ziming et al. [5], where nodes broadcast their position and the user terminal calculates the centroid of the reference point of the received broadcast signal and then takes this as the estimation of the terminal position. A semi-definite programming (SDP) method for collaborative positioning and achieved high robustness in different topological networks was proposed by Monir and Michael [6]. However, they did not consider the sensor user’s own positioning ambiguity problem. A Second-Order Cone Programming (SOCP) model is proposed by Slavisa et al. [7], which can sacrifice a certain positioning accuracy in exchange for a faster calculation speed. However, it did not consider the impact of ranging error on the positioning results. The positioning accuracy decreases rapidly when the ranging error is larger. Fabian studied co-location based on measurement selection sequences and proposed a series of methods to solve the ranging error in co-location but also ignored the positioning error of cooperative nodes [8]. A co-location model approaching the lower limit of positioning error in a non-line-of-sight environment and designed a series of algorithms to solve it [9]. However, this method cannot obtain the global optimal solution, which leads to an increase in the positioning error of the cooperative nodes. A quasi-linear optimization co-location model is proposed by Jiang et al. [10]. The model also considers the effect of coordination and non-line-of-sight errors between cooperative nodes; however, the performance improvement of the algorithm is very limited. Positioning based on factor graphs was proposed by Christian and Simon [11]. It introduced a factor-graph-based positioning algorithm in wireless cellular networks by the base stations and set the base stations as precise nodes, The factor graph was used to obtained the position of mobile nodes. However, it did not consider the effect of the position error and ranging error of base stations and mobile nodes on the cooperative position system. The position error and ranging error are the main limitations of cooperative node localization accuracy. To solve the problem of location ambiguity in distributed cooperative positioning, we propose a cooperative positioning algorithm in this paper under the influence of positioning error and ranging error. The cooperative nodes can measure the distance information and interactive self position information to improve the accuracy of position, which takes into account both the ranging error of cooperative nodes and the fuzzy positioning error of cooperative nodes. We take advantage of factor graph theory to realize the reliability estimation of ranging error and positioning error and integrate the overall least squares theory to achieve a high-accuracy position cooperative position.

In distributed cooperative positioning, because of the large ranging error, the introduction of cooperative positioning creates more positioning errors [12]. Therefore, the distributed cooperative positioning method is proposed in this paper to achieve positioning through the visual distance of the coordinated nodes. The network topology of distributed cooperative positioning G is set as

(1) $$\begin{array}{c} G=(V,E) \end{array}$$

where V represents all cooperative nodes in a distributed cooperative positioning network, and E represents a set of range values between cooperative nodes. The total number of cooperative nodes is *K*, and the position of cooperative node *k* can be expressed as

(2) $$\begin{array}{c} p_{k}={\left[x_{k},y_{k},z_{k}\right]}^{T} \end{array}$$

$x_{k},y_{k}$ and $z_{k}$ represent the position of the x-axis, y-axis, and z-axis of cooperative node *k*. Then, the vector of the position of all cooperative nodes U is represented as

(3) $$\begin{array}{c} U=[p_{1}^{T},p_{2}^{T},\cdots ,p_{K}^{T}]. \end{array}$$

The network topology of distributed cooperative positioning G can be expressed as the product of *K* sets of subgraphs of some cooperative nodes due to factor graph theory [13]. The distributed network topology $G_{k}$ of the cooperative node *k* is set as

(4) $$\begin{array}{c} G_{k}=\left(V_{k},E_{k}\right) \end{array}$$

where $E_{k}$ represents a set of range values between the cooperative node *k* with the other cooperative nodes that have a communication link with cooperative node *k*. The range value between cooperative node *k* and cooperative node *i* can be expressed as

(5) $$\begin{array}{c} r_{ik}=\sqrt{{\left(x_{i}-x_{k}\right)}^{2}+{\left(y_{i}-y_{k}\right)}^{2}+{\left(z_{i}-x_{k}\right)}^{2}}. \end{array}$$

The position of a cooperative node can be obtained by more than three groups of distance equations in a cooperative positioning network [14]. However, the ranging error of distances between cooperating nodes and the position ambiguity of cooperative nodes affects the accuracy of cooperative positioning [15]. To address the ranging error between different cooperative nodes, the paper utilized the range difference function instead of the distance function to abate the ranging error. The range difference $d_{k}^{ij}$ between cooperative node *k* and any other two cooperative nodes i,j is expressed as

(6) $$\begin{array}{c} d_{k}^{ij}=r_{ik}-r_{jk}. \end{array}$$

Combining Equations (3) and (4), Equation (6) can be rewritten as follows:

(7) $$\begin{array}{cc} & \left(x_{i}-x_{j}\right)x_{k}+\left(y_{i}-y_{j}\right)y_{k}+\left(z_{i}-z_{j}\right)z_{k}\\ = & 1/2[\left(x_{i}^{2}+y_{i}^{2}+z_{i}^{2}\right)-\left(x_{j}^{2}+y_{j}^{2}+z_{j}^{2}\right)+\left(r_{i}^{2}-r_{j}^{2}\right)]. \end{array}$$

The belief information is constructed and is transferred between cooperating nodes to obtain the optimal position information of a cooperative node. Belief information is the information describing the mean and standard deviation of the range value between the cooperating nodes and the positioning error of a cooperative node. If cooperative node *i* adjacent to cooperative node *k* has the highest belief information among all cooperative nodes, the belief information of cooperative node *i* is set as the standard belief information for cooperative node *k*; then, the index of cooperative node *i* is set as st. The belief information is computed by factor graph theory in the next part. The range value between cooperative node st and *k* is the standard distance. Then, the distance difference between cooperative node *j* and *k* and cooperative node st and *k* is as follows:

(8) $$\begin{array}{cc} & \left(x_{j}-x_{st}\right)x_{k}+\left(y_{j}-y_{st}\right)y_{k}+\left(z_{j}-z_{st}\right)z_{k}\\ = & 1/2[\left(x_{j}^{2}+y_{j}^{2}+z_{j}^{2}\right)-\left(x_{st}^{2}+y_{st}^{2}+z_{st}^{2}\right)+\left(r_{j}^{2}-r_{st}^{2}\right)]. \end{array}$$

To obtain the position of the cooperative nodes, the aim function can be constructed by multiple sets of Equation (7), and the form of the aim function can be expressed as AX=B. The ith line of matrix X is $\left[x_{i},y_{i},z_{i}\right]$; the ith line of matrix A is $\left[x_{i}-x_{st},y_{i}-y_{st},z_{i}-z_{st}\right]$, and the ith line of matrix B is $1/2[\left(x_{i}^{2}+y_{i}^{2}+z_{i}^{2}\right)-\left(x_{st}^{2}+y_{st}^{2}+z_{st}^{2}\right)+\left(r_{i}^{2}-r_{st}^{2}\right)]$. The matrix can be defined as D=[A,B], and the aim function can be rewritten as

(9) $$D\cdot \left[\begin{array}{c} X\\ -I \end{array}\right]=0.$$

The elements of matrix D are independent with the same distribution. Therefore, the position of cooperative nodes can be obtained by the least minimum square method. However, any cooperative nodes that have a large position error or ranging error will degrade the performance of all the cooperative nodes, so the factor graphs is adopted to abate the effect of the position error and ranging error.

## 2. Factor-Graph-Assisted Distributed Coordination Position Algorithm

Due to the positioning information accuracy variation of cooperative nodes, it is difficult to obtain the optimal position by fixed cooperative nodes [16]. To solve this problem, the factor-graph-assisted element weighting total least squares algorithm is proposed based on parameter estimation theory. The overall optimization position of cooperative nodes is utilized to replace the existing independent optimization of the distributed position cooperative node. First, the cost function of cooperative node *i* is constructed as follows:

(10) $$\begin{array}{c} S_{i}=\sum_{k=1}^{K}\omega_{ki}\left(\right\|p_{k}-p_{i}{\left\|-l_{ki}\right)}^{2} \end{array}$$

where ||.|| means the norm function, *K* represents the total cooperative node, $\omega_{ki}$ represents the weight factor of belief information between cooperative node *k* and *i*, $p_{k}$ and $p_{i}$ represents the position of cooperative node *k* and *i*, and $r_{ki}$ represents the range value between cooperative node *k* and *i*. The overall positioning optimal cost function *S* can be expressed as the sum of cooperative node cost function $S_{i}$.

(11) $$\begin{array}{ccc} S & = & \sum_{i=1}^{K}S_{i}\\ & = & \sum_{i=1}^{K}\sum_{k=1}^{K}\omega_{ki}\left(\right\|p_{k}-p_{i}{\left\|-r_{ki}\right)}^{2}. \end{array}$$

**Figure 1:**
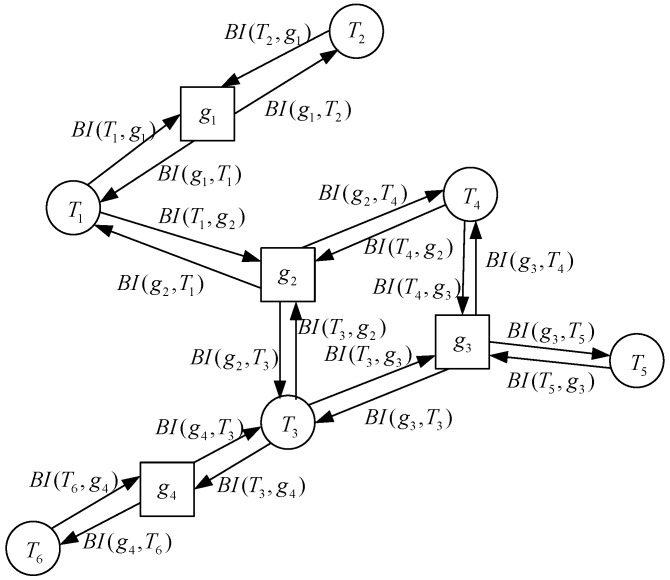
The structure of factor-graph-assisted distributed cooperative positioning.

Factor graph theory has two types of nodes: variable nodes and function nodes. Each edge is connected with a variable node and a function node. In our proposed distributed cooperative position algorithm, the variable node represents the cooperative node, and the function node represents a factor graph local function and achieves nonlinear fusion of belief information in every computation cycle, so there is no link between different variable nodes. The factor graphs method can split a complex multivariate global function into the product of several simple local functions, so the optimal position is obtained by the local function instead of the optimal position of the global function. In product theory of the factor graph, the belief information is transferred between variable nodes and function nodes to obtain the optimal position information of a cooperative node. The structure of distributed cooperative position based on factor graph is shown in Figure 1.

The belief information passed from the cooperative node to the function node is the product of the belief information of all the other neighbor function nodes arriving at the cooperative node. For example, the belief information based on the transfer from the cooperative node $T_{3}$ to the function node $g_{2}$ can be described as

(12) $$\begin{array}{c} BI\left(T_{3},g_{2}\right)=BI\left(g_{3},T_{3}\right)BI\left(g_{4},T_{3}\right) \end{array}$$

where $BI(g_{3},T_{3})$ represents belief information that transfers from function node $g_{3}$ to cooperative node $T_{3}$, and $BI(g_{4},T_{3})$ represents belief information that transfers from function node $g_{4}$ to cooperative node $T_{3}$. The belief information passed from the function node to the cooperative node is the product of all belief information of the other neighbor cooperative nodes connected to the function node and is then multiplied by a local function of the function node. For example, the belief information passed from function node $g_{2}$ to $T_{3}$ can be expressed as

(13) $$\begin{array}{c} BI\left(g_{2},T_{3}\right)=BI\left(T_{1},g_{2}\right)BI\left(T_{4},g_{2}\right)g_{2}\left(T_{1},T_{4}\right) \end{array}$$

where $BI(T_{1},g_{2})$ and $BI(T_{4},g_{2})$ represent belief information that transfers from cooperative nodes $T_{1}$ and $T_{4}$ to function node $g_{2}$. $g_{2}\left(T_{1},T_{4}\right)$ represents the local function of the function node. In this paper, the local function is modeled by a signal propagation decay model and obeys a Gaussian distribution. Then, the belief information of cooperative node $T_{3}$ can be expressed as the product of all belief information connected with cooperative node $T_{3}$, expressed as

(14) $$\begin{array}{c} BI\left(T_{3}\right)=BI\left(g_{2},T_{3}\right)BI\left(g_{3},T_{3}\right)BI\left(g_{4},T_{3}\right). \end{array}$$

The belief information passed from cooperative node $T_{2}$ to $T_{3}$ with the shortest path principle is then expressed as

(15) $$\begin{array}{cc} & BI(T_{2},T_{3})\\ = & BI\left(T_{2}\right)BI\left(T_{2},g_{1}\right)BI\left(g_{1},T_{1}\right)BI\left(T_{1},g_{2}\right)BI\left(g_{2},T_{3}\right). \end{array}$$

Because the maximum value of the belief information in the factor graph is 1, if the number of cooperative nodes is larger, the actual value of the belief information is different by triangulation and leads to larger calculation errors. The normalized weight factor is adopted in our proposed algorithm to abate calculation error and is expressed as

(16) $$\begin{array}{c} w_{ki}=\frac{BI(T_{k},T_{i})}{max(BI\left(T_{k},T_{i}\right))}. \end{array}$$

$w_{ki}$ represents the normalized weight factor of belief information $BI(T_{k},T_{i})$. The optimal position can be obtained by minimizing cost subfunction $S_{i}$ instead of the cost function *S*.

(17) $$\begin{array}{c} \min S=\min\sum_{i=1}^{K}{\left\|\Phi_{i}^{-1/2}\Delta d_{i}\right\|}_{2}^{2}. \end{array}$$

Combining Equations (9) and (10), the cost function can be rewritten as follows:

(18) $$\left(D+\Delta D\right)\left[\begin{array}{c} X\\ -I \end{array}\right]=0$$

where ΔD represents the error of matrix D with zero mean, Δd represents the *i*th line of matrix ΔD, $d_{i}$ represents the *i*th row vector of D and mutual independence, and the covariance matrix $\Phi_{i}$ of $d_{i}$ is expressed as

(19) $$\begin{array}{ccc} \Phi_{i} & = & cov(d_{i})\\ & = & \left[\begin{array}{cc} var(a_{i}) & cov(a_{i},b_{i})\\ cov(b_{i},a_{i}) & var(b_{i}) \end{array}\right]\\ & = & \left[\begin{array}{cc} \Phi_{a_{i}} & \Phi_{a_{i},b_{i}}\\ \Phi_{b_{i},a_{i}} & \Phi_{b_{i}} \end{array}\right] \end{array}$$

where $a_{i}$ and $b_{i}$ represent the *i*th line of matrix A and matrix B. Optimization can be obtained by minimizing the cost function of matrix $\Delta d_{i}$, and the new cost function f(ΔD) is expressed as

(20) $$f\left(\Delta D\right)=\min_{\Delta d_{1},\cdots ,\Delta d_{m}}\sum_{i=1}^{m}{\left\|\Phi_{i}^{-1/2}\Delta d_{i}\right\|}_{2}^{2}.$$

To obtain the optimal position of cooperative nodes, Equation (20) can be rewritten as follows:

(21) $$\begin{array}{ccc} {\mathrm{DX}}_{est}+\Delta {\mathrm{DX}}_{est} & = & 0\\ \gamma_{i}+\Delta d_{i}^{T}X_{est} & = & 0 \end{array}$$

where $X_{est}={\left[X,-I\right]}^{T}$, ${\mathrm{DX}}_{est}$ represents the residual matrix and is expressed as ${\mathrm{DX}}_{est}=\mathrm{AX}-B$. $\gamma_{i}^{T}$ represents the *i*th row of the residual matrix. The optimal position of cooperative node *i* can be expressed as follows by decomposing Equation (21):

(22) $$\begin{array}{ccc} f(\Delta d_{i}) & = & \min_{\Delta d_{i}}\sum_{i=1}^{m}{\left\|\Phi_{i}^{-1/2}\Delta d_{i}\right\|}_{2}^{2}\\ \Delta d_{i}^{T}X_{est} & = & -\gamma_{i}. \end{array}$$

The optimization problem of Equation (22) is a minimum two-norm problem, so the optimal solution of Equation (22) is

(23) $$f\left(\Delta d_{i}\right)=\gamma_{i}^{T}{\left(X_{est}^{T}\Phi_{i}X_{est}\right)}^{-1}\gamma_{i}.$$

Therefore, the optimization problem required by Equation (23) can be expressed as an unconstrained optimization problem. Suppose $Q_{i}\left(X\right)=X_{\mathrm{est}}^{T}\Phi_{i}X_{\mathrm{est}}$; Equation (23) can be rewritten as

(24) $$\begin{array}{ccc} \min S(X) & = & \min\sum_{i=1}^{m}f\left(\Delta D\right)\\ & = & \min\sum_{i=1}^{m}\gamma_{i}^{T}Q_{i}^{-1}\left(X\right)\gamma_{i}. \end{array}$$

The partial derivative of function S(X) with respect to X is

(25) $$\begin{array}{c} S^{\prime }\left(X\right)=2\sum_{i=1}^{m}S_{i}^{\prime }\left(X\right) \end{array}$$

$$\begin{array}{c} S_{i}^{\prime }\left(X\right)=\left(\begin{array}{c} a_{i}^{T}\left(a_{i}X^{\left(n+1\right)}-b_{i}\right)Q_{i}^{-1}\left(X\right)-\left(\Phi_{a_{i}}X^{\left(n+1\right)}-\Phi_{b_{i}}\right)\\ Q_{i}^{-1}\left(X^{\left(n\right)}\right)\gamma_{i}\left(X^{\left(n\right)}\right)\gamma_{i}^{T}\left(X^{\left(n\right)}\right)Q_{i}^{-1}\left(X^{\left(n\right)}\right) \end{array}\right). \end{array}$$

By expanding Equation (19), the following standard linear equation can be obtained:

(26) $$\begin{array}{c} \sum_{i=1}^{m}\left(\frac{a_{i}^{T}a_{i}}{Q_{i}\left(X^{\left(n\right)}\right)}-\Phi_{a_{i}}\frac{\gamma_{i}^{2}\left(X^{\left(n\right)}\right)}{Q_{i}^{2}\left(X^{\left(n\right)}\right)}\right)X^{\left(n+1\right)}\\ =\sum_{i=1}^{m}\left(\frac{a_{i}^{T}b_{i}}{Q_{i}\left(X^{\left(n\right)}\right)}-\Phi_{b_{i}}\frac{\gamma_{i}^{2}\left(X^{\left(n\right)}\right)}{Q_{i}^{2}\left(X^{\left(n\right)}\right)}\right). \end{array}$$

The optimal position of cooperative nodes $X^{\left(n+1\right)}$ can then be computed by the iterated operation in Equation (26). When $\|X^{\left(n+1\right)}-X^{\left(n\right)}\left\|/\right\|X^{\left(n\right)}\|<\theta$, the position results are stable and $X^{\left(n+1\right)}$ is the global optimal position of the cooperative nodes estimated from the global conditions. θ is the judgment threshold and is determined by the error fluctuation.

## 3. Simulation Results and Analysis

### 3.1. Ranging Error Performance Analysis

**Figure 2:**
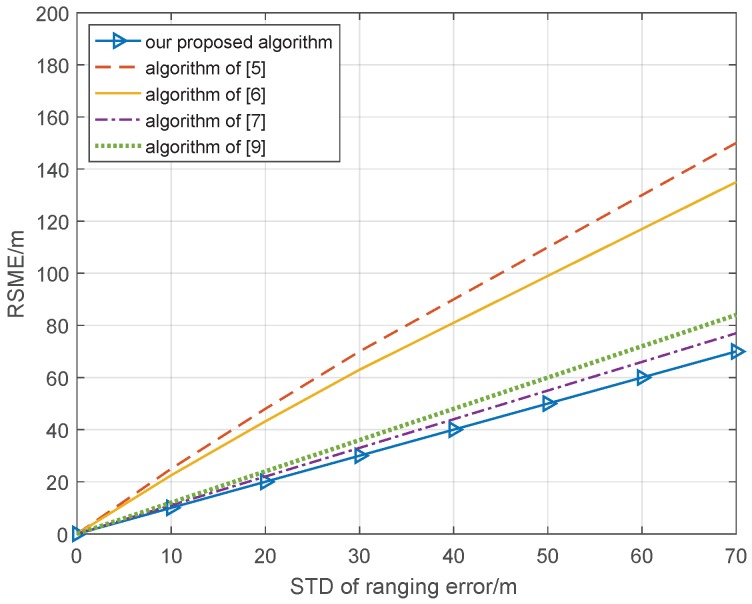
Relationship cooperative position error and ranging error with ideal position condition.

In the cooperative position system, positioning accuracy is mainly affected by the positioning error of the cooperative node and the ranging error, which is measured between cooperative nodes. In the first part, the ranging error is simulated with the ideal position condition, where the standard deviation of the positioning errors of cooperative nodes is 0 m. Our proposed algorithm is compared with the cooperative position method based on distance measurement assistance in [5], semi-definite collaborative positioning method in [6], the second-order cone optimized co-location method in [7], and the quasi-linear programming co-location method in [9]; the radius of the network topology is 5 km; the range value between cooperative nodes is independent and is obtained via Wifi; the position of cooperative nodes is obtained by GPS and the data frequency of ranging and position are both 100 Hz. In each calculation, the cooperative node is stable in network topology. The framework of the cooperative network topology is shown in Figure 1. The standard deviation (STD) of the ranging error is from 0 to 70 m, and the simulation result of 1000 Monte Carlo is shown in Figure 2.

**Figure 3:**
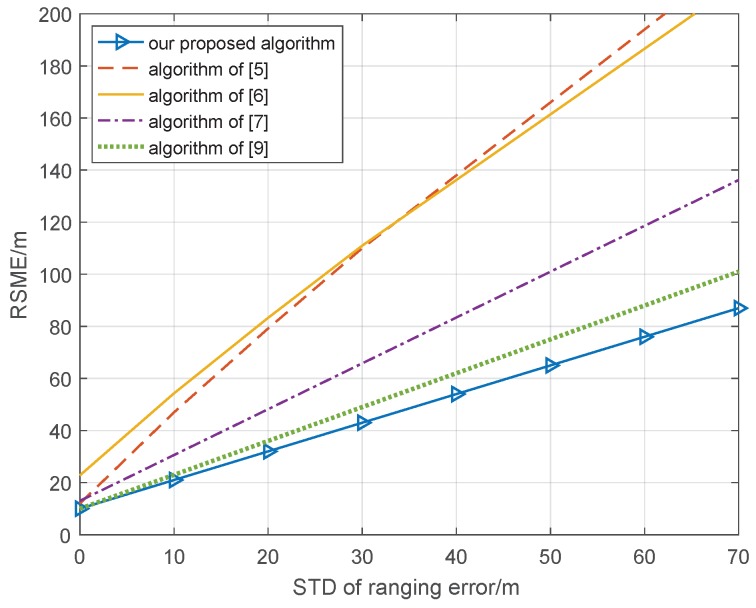
Relationship cooperative position error and ranging error with STD of positioning errors of cooperative nodes of 10 m.

From Figure 2, we can see that the RSME (Root Mean Square Error) of all cooperative position methods becomes larger with the increase in standard deviation of the ranging error. However, the methods in [5,6] exhibit faster performance degradation than the other methods; when the STD of the ranging error is 40 m, the RSME of the cooperative position is 90 and 80 m, respectively. However, the methods in [7] and [9] as well as our proposed algorithm can effectively prevent degradation of the RSME; when the STD of the ranging error is 40 m, the RSME of the cooperative position is 45, 50, and 40 m, respectively. This shows that our proposed algorithm can effectively reduce the impact of the ranging error on the cooperative position. Because it is difficult for a real cooperative position environment to maintain a standard deviation of cooperative node position error of 0 m, the STD of the positioning errors of the cooperative nodes is set to 10 m, which depends on the positioning accuracy of the GNSS system, and the other condition is the same; the simulation result of 1000 Monte Carlo is shown in Figure 3.

From Figure 3, we can see that, because the cooperative nodes have a position error, the RSME of the methods in [5,6] degrade quickly, and it is difficult to achieve performance improvement with respect to the cooperative position. Compared with the result in Figure 2, the RSME of the methods in [7,9] also have larger degradation; when the STD of the ranging error is 40 m, the RSME of the cooperative position is 84 and 63 m, respectively. This means the position error of the cooperative nodes have a larger effect and the methods in [7,9] ignore the position error with difficulty. However, the RSME of our proposed algorithm is only 55 m, and this is better than those of the other algorithms. The factor map theory is utilized to estimate the belief information; then, combining the overall least squares method to abate the effect of cooperative node position error helps to improve the performance of the cooperative position system.

### 3.2. Cooperative Node Positioning Error Performance Analysis

**Figure 4:**
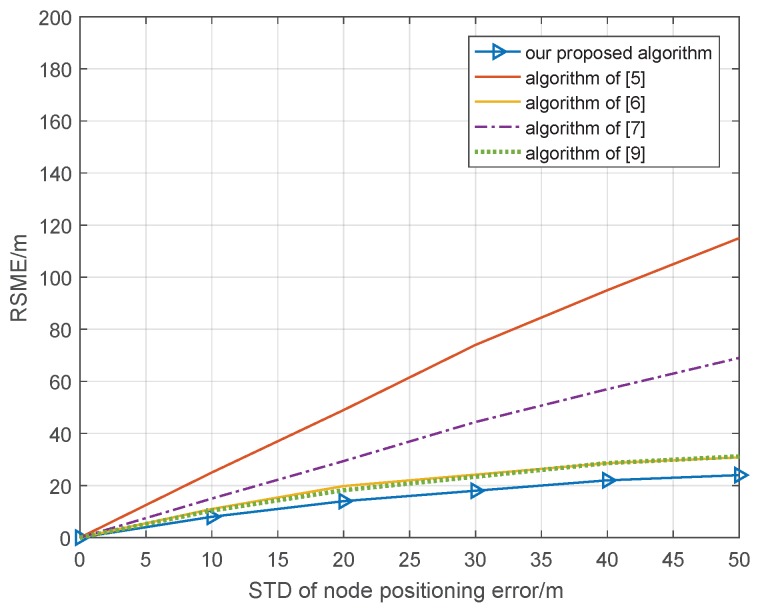
Relationship cooperative position error and node positioning error with ideal position condition.

In the cooperative position system, in addition to the ranging error, the position error of cooperative nodes will have a significant impact on the accuracy of the cooperative position system. In the second part, the positioning errors of cooperative nodes are simulated with the ideal range condition, where the standard deviation of the ranging error is 0 m; our proposed algorithm is compared with the cooperative position method based on distance measurement assistance in [5], the semidefinite collaborative positioning method in [6], the second-order cone optimized co-location method in [7], and the quasi-linear programming co-location method in [9]; the other simulation condition is the same as that in Section 3.1, and the simulation result of 1000 Monte Carlo is shown in Figure 4.

**Figure 5:**
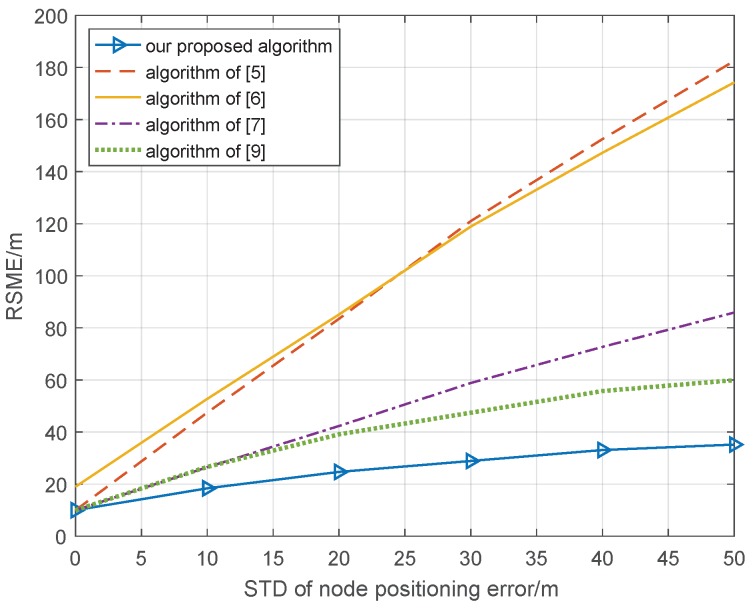
Relationship cooperative position error and node positioning error, wherethe STD of ranging error is 10 m.

In Figure 4, we can see that the RSME of all cooperative position methods will become larger with an increasing standard deviation of the node positioning error. However, the methods in [5,7] exhibit faster degradation than do other methods; when the STD of the node positioning error is 30 m, the RSME of the cooperative position is 75 and 45 m, respectively. Our proposed algorithm and the methods proposed by [6,9] can remove the effect of node position error and improve the performance of the cooperative position; when the STD of the node position error is 30 m, the RSME of the cooperative position is 22, 22, and 18 m, respectively. As with the analysis of the ranging error, it is difficult for the real cooperative position environment to maintain a standard deviation of the ranging error of 0 m, so we set the STD of the ranging error to be 10 m and repeat the simulation again; the other condition is the same as in Figure 4, and the simulation result of 1000 Monte Carlo is shown in Figure 5.

In Figure 5, we can see that, due to the effect of the ranging error, the RSME of the proposed algorithms in [5,6] degrade quickly and are unable to meet the requirements of the cooperative position system. Compared with the results in Figure 4, due to the effect of ranging error, the RSME of the proposed algorithms in [5,6] degrade quickly and are unable to meet the requirements of the cooperative position system. Compared with the results in Figure 4, the proposed algorithms in [7,9] are unable to completely eliminate the ranging error, so the RSME is worse; when the STD of the node position error is 30 m, the RSME of the cooperative position is 60 and 50 m, respectively. However, the RSME of our proposed algorithm is only 28 m when the STD of the node position error is 30 m and exhibits great improvement over the other cooperative position algorithms; our algorithm utilized the factor graph to establish cooperative node belief information, and data fusion was then performed, whereby the cooperative position system adopts the cooperative node that has a high value of belief information. The global cost function is thus constructed to obtain the global optimum of all cooperative nodes, and the effect of the positioning error and ranging error on the accuracy of the cooperative position can be effectively reduced.

### 3.3. Comparison of Convergence Rates

**Figure 6:**
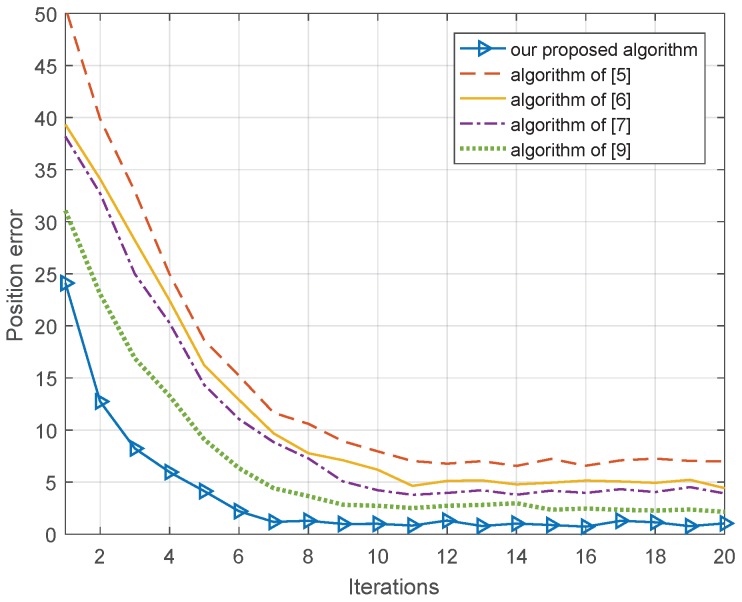
Convergence rates.

In the cooperative position system, nodes have both moving and static states, so the convergence speed of the algorithm will affect the actual performance of the algorithm; a faster convergence rate will bring better positioning performance. Therefore, the STD of the ranging error and nodes positioning error is 1 m, a value that depends on the existing GNSS position accuracy of cooperative nodes. Our proposed algorithm is compared with the cooperative position algorithms proposed in [5,6,7,9]; the simulation result of 1000 Monte Carlo is shown in Figure 6.

In Figure 6, we can see that the convergence speed of [5] is the slowest, requiring 12 iterations to complete the convergence, and the cooperative position error reaches 7 m due to the large influence of the cooperative node positioning error and ranging error. The algorithms in [6,7] require 11 iterations to complete the convergence, and the cooperative position error reaches 5 m and 4.6 m; the algorithms in [6,7] ignore the effect of the ranging error and node positioning error. Our proposed algorithm and the proposed algorithm in [9] take into account both the ranging error and position error of the cooperative node, so the cooperative position errors reach 1 m and 3 m, respectively. The convergence speed of our proposed algorithm is the fastest among all the algorithms; the algorithm of this paper utilized the adaptive belief information function of cooperative nodes based on factor graph theory to select the nodes that have better node position accuracy and smaller ranging error, so our proposed algorithm can remove the impact of positioning error and ranging error quickly and improve the convergence rate.

### 3.4. Effect of Cooperative Nodes Mutation Error

**Figure 7:**
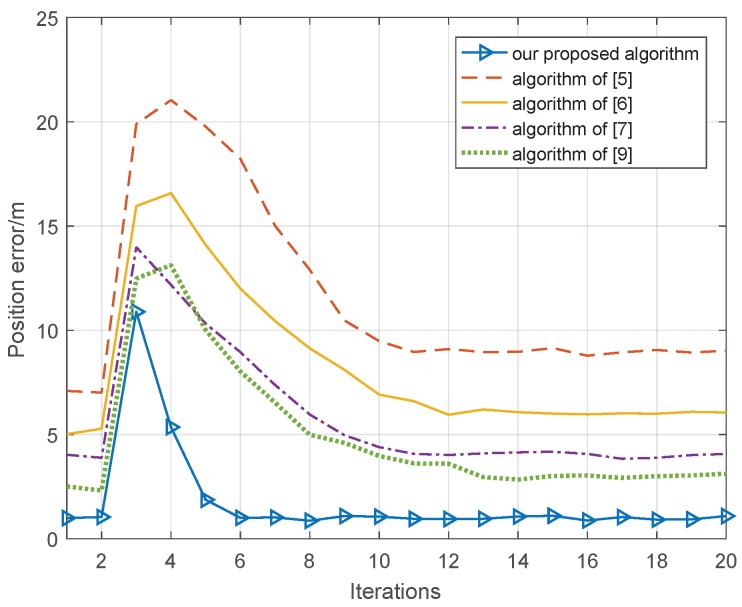
Cooperative position performance with mutation error.

In the cooperative position system, the GNSS position result of the cooperative nodes depends on the quality of the GNSS signal. Satellite signal interference, signal scattering and deceptive signals will affect the GNSS signal and lead to mutation error, which will degrade the the position performance of cooperative nodes, so the cooperative position algorithm should reduce the influence of mutation error on the cooperative positioning system. Under a standard deviation of the ranging error and node positioning error of 1 m, three nodes in the cooperative position network are added to the mutation positioning error in the third moment after the cooperative positioning network is stable; the simulation result of 1000 Monte Carlo is shown in Figure 7.

In Figure 7, we can see that the algorithm proposed in [5] requires eight iterations to achieve the re-convergence of the cooperative position system, and the cooperative position error exhibits a drop of about 2 m. The algorithms from [6,7,9] require 8, 7, and 10 iterations, respectively, to achieve re-convergence, and it is difficult for all of the cooperative position algorithms to adapt to moving nodes. Our proposed algorithm just requires three iterations to achieve re-convergence, and the accuracy position of the cooperative node is stable. This is mainly because the decomposition of the factor graph is utilized to directly obtain the optimal positioning result of the cooperative node in the whole cooperative network, and the belief information of the cooperative node can avoid cooperative nodes that have a mutation error selected by the cooperative position. Thus, our proposed algorithm can eliminate the influence of mutation error on the cooperative node.

## 4. Conclusions

In response to the bottleneck in improvements to positioning accuracy in the existing navigation and positioning technology, the cooperative position algorithm can further improve positioning accuracy based on the interactive position information of the cooperative node. However, the ranging error and node positioning error have a greater impact on the accuracy of a cooperative positioning network and even reduce positioning accuracy. Our proposed factor-graph-assisted distributed cooperative position algorithm establishes the corresponding belief information model of the cooperative node by the ranging error and node position error and combines the total least squares method to obtain the optimal position of the cooperative position network. It can effectively restrain the influence of ranging error and node positioning error on the whole cooperative positioning system. This paper compares our proposed algorithm with the existing algorithms in terms of ranging error, cooperative node positioning error, convergence rate, and mutation error; the simulation results show that the positioning accuracy of our proposed algorithm is improved by 30–60%. In terms of convergence rate, our proposed algorithm utilizes the sum-product principle of the factor graph to achieve faster convergence than the other cooperative position algorithms. Importantly, when the cooperative node itself has a mutation error, our algorithm can quickly eliminate the effect of the mutation error on the entire coordinated positioning network. Our method has good application value in the field of navigation and positioning.
